# SOX14 activates the p53 signaling pathway and induces apoptosis in a cervical carcinoma cell line

**DOI:** 10.1371/journal.pone.0184686

**Published:** 2017-09-19

**Authors:** Danijela Stanisavljevic, Isidora Petrovic, Vladanka Vukovic, Marija Schwirtlich, Marija Gredic, Milena Stevanovic, Jelena Popovic

**Affiliations:** 1 Institute of Molecular Genetics and Genetic Engineering, University of Belgrade, Belgrade, Serbia; 2 University of Belgrade, Faculty of Biology, Belgrade, Serbia; 3 Serbian Academy of Sciences and Arts, Belgrade, Serbia; Virginia Commonwealth University, UNITED STATES

## Abstract

SOX14 is a member of the SOX family of transcription factors mainly involved in the regulation of neural development. Recently, it became evident that *SOX14* is one of four hypermethylated genes in cervical carcinoma, considered as a tumor suppressor candidate in this type of malignancy. In this paper we elucidated the role of SOX14 in the regulation of malignant properties of cervical carcinoma cells *in vitro*. Functional analysis performed in HeLa cells revealed that SOX14 overexpression decreased viability and promoted apoptosis through altering the expression of apoptosis related genes. Our results demonstrated that overexpression of SOX14 initiated accumulation of p53, demonstrating potential cross-talk between SOX14 and the p53 signaling pathway. Further analysis unambiguously showed that SOX14 triggered posttranslational modification of p53 protein, as detected by the significantly increased level of phospho-p53 (Ser-15) in SOX14-overexpressing HeLa cells. Moreover, the obtained results revealed that SOX14 activated p53 protein, which was confirmed by elevated p21^Waf1/Cip1^, a well known target gene of p53. This study advances our understanding about the role of SOX14 and might explain the molecular mechanism by which this transcription factor could exert tumor suppressor properties in cervical carcinoma.

## Introduction

*SOX* family of genes encode for transcription factors that are conserved across species and participate in important developmental processes [1–3]. In addition, members of this group of genes are involved in malignant phenotypes through their ability to regulate numerous cancer hallmarks, including cell proliferation, apoptosis, survival, invasion, migration, stemness, differentiation, senescence and angiogenesis [4]. Almost all members of the SOX family have been found to be deregulated in a wide variety of tumors, where they have either oncogenic or tumor suppressor properties [4].

SOX14 transcription factor is mainly involved in the regulation of neural development [5,6]. Although its pivotal role is associated with developmental processes, there are several studies suggesting that SOX14 is involved in cancerogenesis, but its significance has not been clearly determined. *SOX14* expression studies revealed that this gene is downregulated in MCF7 breast adenocarcinoma cells through a still unexplained mechanism [7]. Genome-wide analysis of aberrant DNA methylation has shown that *SOX14* is one of the genes methylated in patients with chronic lymphocytic leukemia [8]. Recently, it became evident that SOX14 is involved in cervical cancerogenesis, but there are conflicting data regarding its function in cells derived from this type of neoplasm. One group showed that SOX14 can promote proliferation and invasion capacity of cervical cancer cells by activating the Wnt/β-catenin pathway [9]. However, others have revealed that *SOX14* gene is one of four hypermethylated markers applicable for screening of both adeno- and squamous-cell cervical carcinoma and is unmethylated in normal tissue [10]. In cervical carcinoma samples it has been shown that the genomic region where *SOX14* is located (chromosome 3q23) encompasses several tumor suppressor genes [11].

Having in mind the inconsistent data regarding the function of SOX14 in cervical carcinoma, our aim was to evaluate its role in the regulation of malignant properties of cervical carcinoma cells *in vitro*. This included analysis of methylation status and its regulatory influence on cell migration, invasion, viability, apoptosis and the cell cycle. Our analysis revealed that SOX14 induced apoptosis, changed the cell cycle distribution and inhibited the viability of cervical cancer cells *in vitro*. Moreover, we have demonstrated that SOX14 overexpression stabilized p53 and induced p21^Waf1/Cip1^ protein expression, suggesting that SOX14 alone is able to upregulate the p53 signaling pathway. Furthermore, we have revealed that SOX14 induced p53 stabilization by increasing the amount of phosphorylated p53 at position Serine 15. The results presented here advance our understanding of SOX14 as a novel tumor suppressor candidate in cervical cancer cells.

## Material and methods

### Cell culture and transfection

HeLa (ATCC^®^, CCL-2) cells were maintained in Dulbecco's Modified Eagle's medium (DMEM) supplemented with 10% fetal bovine serum (FBS) and 1% non-essential amino acids (NEAA) (all from Invitrogen^™^, NY, USA), at 37°C in 5% CO_2_. Transfection of HeLa cells was carried out as previously described [6]. NT2/D1 cells, kindly provided by Prof. P.W. Andrews (University of Sheffield, UK) were maintained in Dulbecco's Modified Eagle's medium (DMEM) supplemented with 10% fetal bovine serum (FBS) 4500 mg/L glucose, 2 mmol/L L-glutamine and penicillin/streptomycin (all from Invitrogen^™^, NY, USA), at 37°C in 10% CO_2_ as previously described [12]. For 5-azacytidine treatment (5-azaC, Acros Organics, Belgium), cells were grown for 72 h in 5 μmol/L 5-azaC.

### Generation of expression constructs

Fragments F1R3 and F1R4 encompassing +196/+529 and +196/+676 of the human *SOX14* sequence respectively (333 and 480 bp in length, 279 and 426 bp of the coding sequence respectively) were amplified by PCR from genomic clone SOX14P32.2XbaI [13], using primers F1 5´-CTCGTCTGCAGAACCCTTGCAC-3´ (forward), R3–5´-GCTCAAGAAGGACAGGTATGTC-3´ (reverse) and R4 5´-CTCCCTGCTGGACCCCGCGC-3´ (reverse). The PCR reaction was performed using KAPA 2G Fast HotStart Ready Mix (Kapa Biosystems, MA, USA) according to the manufacturer's protocol. The PCR products were eluted from agarose gel and cloned into pJET1.2 vector using a CloneJET PCR Cloning Kit (Fermentas, Thermo Fisher Scientific, USA). The selected clones were fully sequenced in order to verify that no mutations were introduced by PCR. Using *Bgl*II digestion, the F1R3 or F1R4 fragment was released from pJET1.2 and then subcloned into pcDNA3.1 vector using *BamH*I compatible ends, generating pcDNA3.1SOX14DN1 (SOX14DN1) and pcDNA3.1SOX14DN2 (SOX14DN2) constructs respectively.

### Transfection assays

For immunocytochemical analysis of phospho-p53, cells were cultured in 24 well dishes and empty vector, SOX14wt or DN1 were co-transfected with pEGFP-C1 (Clontech Laboratories, Mountain View, CA, USA). For the luciferase assay HeLa cells were co-transfected with pcDNA3.1, pcDNA3.1SOX14 (SOX14wt) [6], SOX14DN1 or SOX14DN2, as previously described [6]. For the luciferase assay with p53 and p21 promoters, HeLa cells were seeded in 24-well plates and grown for 1 day until they reached approximately 90% confluency. Cells were co-transfected with 500 ng of PGL2-356bp (gift from Wafik El-Deiry, Addgene plasmid # 16292), pGL2-200bp (gift from Wafik El-Deiry, Addgene plasmid # 16293), PGL2- 100bp (gift from Wafik El-Deiry, Addgene plasmid # 16294), PGL2- 50bp (gift from Wafik El-Deiry, Addgene plasmid # 16295), p21-Luc, and 300 ng of pcDNA3.1, SOX14wt, SOX14DN1 or p53wt using Lipofectamine^®^ 2000 and 3000 reagent (Invitrogen^™^, USA) or PEI transfection reagent (Polyethyleneimine “MAX”, Polysciences.Inc, Cat No 24765) according to the manufacturer's protocol. Transfection efficiency was normalized with 5 ng of pRLSV40 plasmid (Promega, USA). Cells were harvested and lysed in Reporter Lysis Buffer (Promega, USA) 24 h after transfection and extracts were assayed for luciferase activity using a Dual-luciferase^®^ Reporter Assay System (Promega, USA).

### RT-PCR and qRT-PCR

Total RNA was isolated, treated with DNase I and subjected to cDNA synthesis as described earlier [6]. The synthesized cDNAs were used as templates for amplification with primers specific for *SOX14* and *GAPDH*. Primers for *SOX14* amplification were as follows: 5´-CTCGTCTGCAGAACCCTTGCAC-3´ (forward), 5´-GCTCAAGAAGGACAGGTATGTC-3´ (reverse). *GAPDH* was amplified with 5′-GGACCTGACCTGCCGTCTAG-3′ (forward) and 5′-CCACCACCCTGTTGCTGTAG-3′ (reverse) to control for equivalent amounts of cDNA per reaction. RT-PCRs were performed in 20 μl reaction mixtures using KAPA 2G Fast HotStart Ready Mix (Kapa Biosystems, MA, USA) according to the manufacturer's protocol. The relative level of *SOX14* expression was presented as a percentage of mRNA expression in HeLa cells transfected with empty vector (mock).

For quantitative PCR analysis, cDNAs were subjected to real time PCR using Power SYBR Green PCR Master Mix (Applied Biosystems^®^) in 7500 Real Time PCR Systems (Applied Biosystems^®^). Primers for *TP53* amplification were as follows: 5'-CCCCTCCTGGCCCCTGTCATCTTC-3' (forward) and 5'-GCAGCGCCTCACAACCTCCGTCAT-3' (reverse). *Bax* was amplified using primers 5’-TGGCAGCTGACATGTTTTCTGAC-3’ (forward) and 5’-TCACCCAACCACCCTGGTCTT-3’ (reverse), while for *Bcl*2 amplification we used 5’-TCGCCCTGTGGATGACTGA-3’ (forward) and 5’- CAGAGACAGCCAGGAGAAATC-3’ (reverse) primers. *CDKN1A*/p21^Waf1/Cip1^ was amplified using primers 5'-GACACCACTGGAGGGTGACT-3' (forward) and 5'-CAGGTCCACATGGTCTTCCT-3' (reverse). *GAPDH* was amplified using primer sets, as mentioned above. All samples were analyzed in triplicate and mean values recorded. The relative level of each analyzed gene was calculated using a comparative quantification algorithm where the resulting ΔΔCt value was incorporated to determine the fold difference in expression (2−ΔΔCt).

### Methylation-specific PCR

Direct sodium-bisulfite conversion of DNA from HeLa and NT2/D1 cells was performed using EZ DNA Methylation-Direct^™^ Kit (Zymo Research Corporation, CA, USA). For conversion 10^5^ cells were used and the protocol provided by the manufacturer was strictly followed. The converted DNA sample was employed as the template for PCR amplification using KAPA 2G Fast HotStart Ready Mix (Kapa Biosystems, MA, USA) and cycling conditions 95°C for 5 minutes, 95°C for 30 seconds, 60°C for 20 seconds, 72°C for 2 minutes, for 35 cycles. The following primers were used for amplification of the methylated (M) versus unmethylated (U) *SOX14* gene promoter sequence:

M1:
     5'-TAAGGGTTTATTAATTAGGGTTCGA-3'
     5'-ACGATACTTTAACAATATTCTCCCG-3'
U1:
     5'-TAAGGGTTTATTAATTAGGGTTTGA-3'
     5'-AATACTTTAACAATATTCTCCCAAA-3'
M2:
     5'-ATAGTGTTCGAGATAATGTGGAATC-3'
     5'-AAAAAACGCGACTAATAAAAACG-3'
U2:
     5'-AGTGTTTGAGATAATGTGGAATTGA-3'
     5'-CAAAAAACACAACTAATAAAAACAAA-3'

The obtained products were separated by electrophoresis on 2% agarose gel and visualized using ethidium bromide staining.

### Western blot

Western blot analysis was performed as previously described [6]. For Western blot analysis of phospho-p53, we added phosphatase inhibitors in lysis buffer (PhosSTOP, Roche). The primary antibodies were: rabbit polyclonal antibody against SOX14 (Abcam, Cambridge, UK, ab149047, diluted 1:400), mouse monoclonal anti-α-Tubulin (Calbiochem, MA, USA, CP06, diluted 1:30000), mouse monoclonal antibody against GAPDH (Abcam, Cambridge, UK, ab9484, diluted 1:5000), mouse monoclonal antibody against cleaved PARP (Cell Signaling, Technology, Danvers, MA, USA, Asp214, diluted 1:1000), mouse monoclonal antibody against p53 (DO-1) (Santa Cruz Biotechnology, Texas, USA, sc-126X, diluted 1:1000), rabbit monoclonal antibody against p21^Waf1/Cip1^ (Cell Signaling, Technology, Danvers, MA, USA, 2947, diluted 1:1000) and rabbit antibody against phospho-p53 (Cell Signaling, Technology, Danvers, MA, USA, diluted 1:1000). Secondary antibodies used for Western blot were: horseradish peroxidase-conjugated anti-mouse and anti-rabbit IgG (Amersham Biosciences, NJ, USA, diluted 1:10000 or Active motif, La Hulpe, Belgium, 1:5000). Immunoreactive bands were detected by chemiluminescence (Immobilion substrate, Millipore, MA, USA). The density of protein bands on Western blots was quantified using ImageJ software.

### Immunocytochemistry (ICC)

Twenty-four hours following the transfection, cells were fixed in 4% paraformaldehyde (PFA, Merck, Germany) for 20 minutes at RT. Cells were permeabilized in 0.1% Triton X-100 and blocked in 1% bovine serum albumin (BSA, Sigma-Aldrich, MO, USA), 10% normal goat serum, 0.1% Triton X-100 in PBS for 1 h at RT. Primary antibodies were diluted in PBS containing 1% BSA, 0.1% Triton X-100 and incubated overnight at 4°C as follows: rabbit polyclonal anti-SOX14 (Abcam, Cambridge, UK, ab149047, diluted 1:200), mouse monoclonal anti-p53 (Santa Cruz Biotechnology, sc-126X, diluted 1:200), rabbit antibody against phospho-p53 (Cell Signaling, Technology, Danvers, MA, USA, diluted 1:50) or rabbit monoclonal antibody against p21^Waf1/Cip1^ (Cell Signaling, Technology, Danvers, MA, USA, 2947, diluted 1:50). Coverslips were washed 3×10 minutes in 0.1% Triton X-100 in PBS (PBT) and incubated with anti-rabbit guinea-pig secondary antibody conjugated with Alexa Fluor^®^ 488 (Invitrogen^™^, diluted 1:500 in 1% BSA-PBT) or Alexa Fluor^®^ 594 (Invitrogen^™^, diluted 1∶500 in 1% BSA-PBT) for 60 minutes at room temperature (RT). The p53 and phospho-p53 antibodies were first labeled with biotinylated goat anti-mouse or anti-rabbit IgG (Vector, Burlingame, CA, USA) for 1 h at RT in 1% BSA in PBT followed by DyLight^®^594-streptavidin (Vector, Burlingame, CA, USA diluted 1:1000) diluted in PBS for 1 h at RT.

Nuclei were stained with 0.1 mg/ml diamino phenylindole (DAPI; Sigma-Aldrich, MO, USA). Samples were viewed and images were taken using a Leica TCS SP8 confocal microscope and Leica Microsystems LAS AF-TCS SP8 software (Leica Microsystems).

### Apoptosis assay

Cells were transfected with empty vector, SOX14wt or SOX14DN1 and 24 h after transfection the cells were washed twice with cold PBS, resuspended in 1× Annexin binding buffer at a final number of 1 × 10^6^ cells/ml. 5 μl of Annexin V (Annexin V, Alexa Fluor^®^ 488 conjugate, Invitrogen^™^) and 5 μl of propidium iodide (PI—Invitrogen) were added. The cells were gently mixed, incubated for 10 minutes in the dark at RT and examined in a BD LSRFortessa^™^ cell analyzer (BD Biosciences, USA). The flow cytometer collected 100.000 events, which were analyzed using FACSDiva Software and FCS Express, Version 3, Research Edition.

### MTS assay

The MTS viability and proliferation test was performed 24 h after transfection of HeLa cells with empty pcDNA3.1, SOX14wt or SOX14DN1 using Promega CellTiter 96^®^ AQueous One Solution Cell Proliferation Assay (Promega, USA) according to the manufacturer’s instruction.

### Cell cycle analysis

Cells were trypsinized and washed with cold 1xPBS twice. Opened tubes with 10^6^ cells were placed in 1 ml of cold 1xPBS, vortexed and during vortexing 4 ml of chilled ethanol was added dropwise to a final concentration of 80%. The cells were incubated in ethanol for 30 minutes and stained with DAPI at 0.01mg/ml final concentration, followed by examination in a BD LSRFortessa^™^ cell analyzer (BD Biosciences, USA). The flow cytometer collected 100.000 events, which were evaluated using FACSDiva Software and FCS Express, Version 3, Research Edition.

### Wound-healing assay

A total of 3x10^5^ cells were plated in 35 mm^2^ dishes and transfected with pcDNA3.1, SOX14wt or SOX14DN1 1 day before the experiment. Cell migration was analyzed as previously described [14]. Cell migration rate was recorded by capturing two different parts of the wounded area, at two time points (0 h and 5 h after wounding).

### Transwell migration and invasion assay

Transwell polycarbonate membrane cell culture inserts with 8.0 μm pores (Corning, NY, USA) were uncoated for the migration assay or coated with 50 μl Matrigel Basement Membrane Matrix (Corning, NY, USA) (at the final concentration 1μg/μl) for the invasion assay. For both assays cells were transiently transfected 24 h before seeding with SOX14wt or the corresponding empty vector as a control. Both assays were further performed as previously described [14].

## Results

### *SOX14* is methylated in the cervical cancer cell line

**Fig 1:**
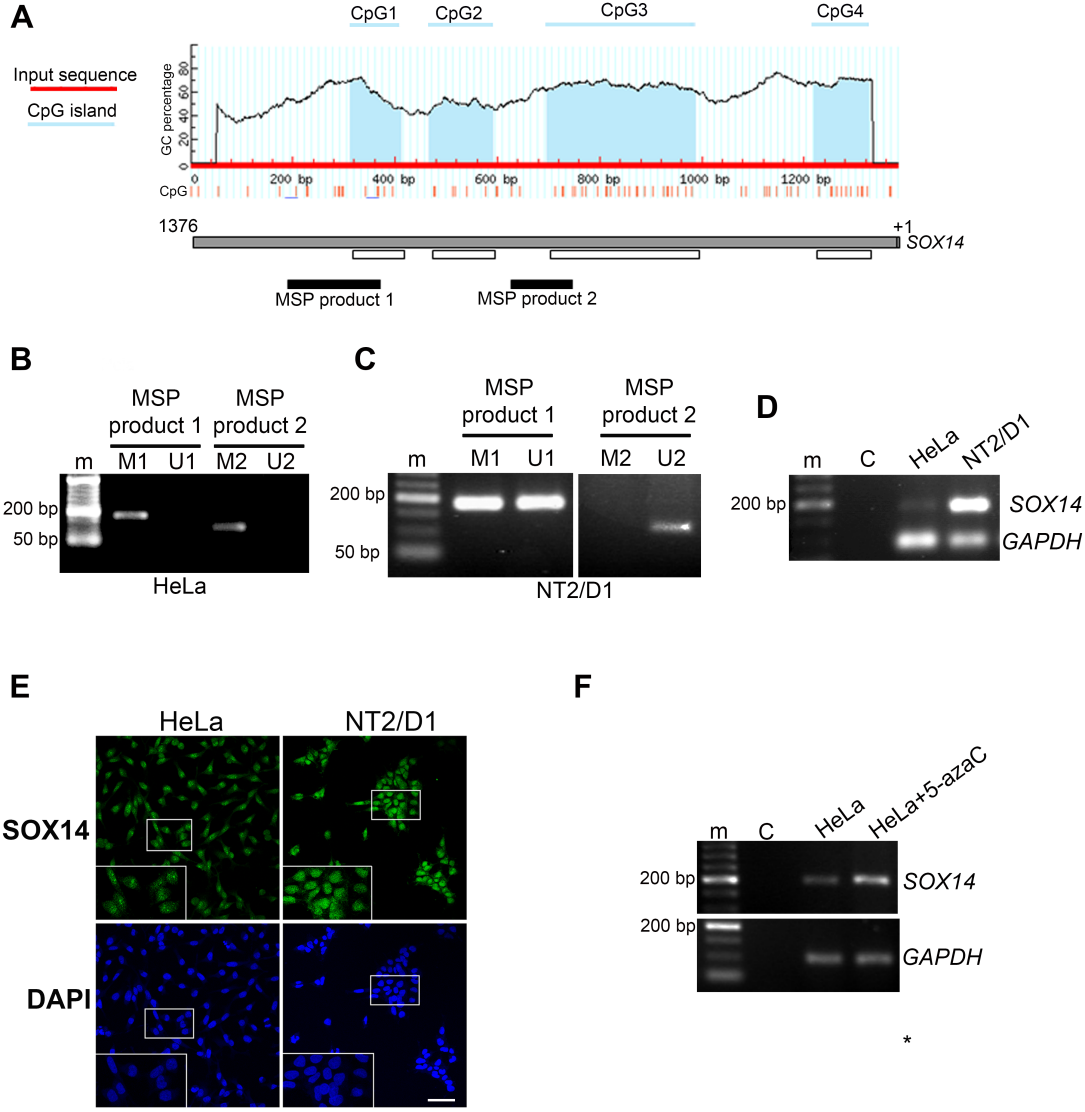
*SOX14* methylation status and expression analysis in the HeLa cell line. **A**- Schematic illustration of *SOX14* promoter and positions of CpG islands 1, 2, 3 and 4 (presented in blue); positions of methylation specific PCR products 1 and 2 (MSP1 and MSP2) are shown in black lines. **B**- Methylation analysis of the *SOX14* promoter by methylation specific PCR (MSP) in HeLa cells. **C**- Methylation analysis of the *SOX14* promoter by MSP in NT2/D1 cells. M- primer pairs that anneal only to sequences that are methylated before bisulfite treatment; U—primer pairs that anneal only to sequences that are unmethylated. The product corresponding to MSP1 is 183 bp in length, while that corresponding to MSP2 is 118 bp in length. **D**- RT- PCR analysis of *SOX14* expression in HeLa and NT2D1 cells. *GAPDH* was used as a loading control. Three independent experiments were performed and one representative PCR is shown. NT2/D1 cells were used as a positive control for *SOX14* expression. m- DNA ladder, C- PCR negative control. **E**–Immunocytochemical analysis of SOX14 expression in HeLa and NT2/D1 cells. NT2/D1 cells were used as the positive control for SOX14 expression. Scale bar: 50 μm. **F**–Expression of *SOX14* following treatment with 5-azaC. HeLa cells were treated with 5μM 5-azaC for 72 h. Total RNA was subjected to RT-PCR in order to analyze *SOX14* mRNA expression. *GAPDH* was used for normalization. Three independent experiments were performed, and one representative PCR is shown. m- DNA ladder, C- PCR negative control.

Recently it has been shown that human *SOX14* is methylated in cervical cancer samples from patients [10,11]. Our first step was to evaluate its methylation status in HeLa cells, a commonly used cervical cancer model system derived from a cervical adenocarcinoma. For prediction of the CpG islands, the human *SOX14* promoter sequence 1382 bp in length [15] was examined using the web-based MethPrimer tool [16]. Conducted using default criteria for CpG island prediction, this analysis identified the presence of four CpG islands within the human *SOX14* promoter designated as CpG islands 1 to 4 (Fig 1A). DNA extracted from HeLa and NT2/D1 cells was subjected to bisulfite conversion followed by methylation-specific PCR (MSP) reactions. Specific PCR products were obtained for CpG islands 1 and 3. As shown in Fig 1B, products obtained with primer sets corresponding to methylated DNA were detected in the HeLa cells, while no products with primer sets corresponding to unmethylated DNA were obtained for either CpG island. In contrast, for NT2/D1 cells we obtained PCR products with primer sets corresponding to both methylated and unmethylated CpG island 1, while for CpG island 3 only an unmethylated product was detected (Fig 1C). These results demonstrated that *SOX14* promoter is methylated in the cervical cancer cell line, particularly in regions encompassed by CpG islands 1 and 3, while in the embryonal carcinoma NT2/D1 cell line the *SOX14* promoter has a lower methylation rate. In accordance with these results, we detected weaker expression of *SOX14* in HeLa cells compared to that for NT2/D1, using both RT-PCR and immunocytochemistry (Fig 1D and 1E). Furthermore, following treatment of HeLa cells with the DNA demethylation agent, 5-azacytidine (5-azaC) [17], we observed a significant increase in *SOX14* mRNA level (Fig 1F). These results showed that induced demethylation activated transcription of SOX14, which further underlines our finding that *SOX14* is methylated in HeLa cells.

### Construction and characterization of novel human SOX14 dominant negative mutants

**Fig 2:**
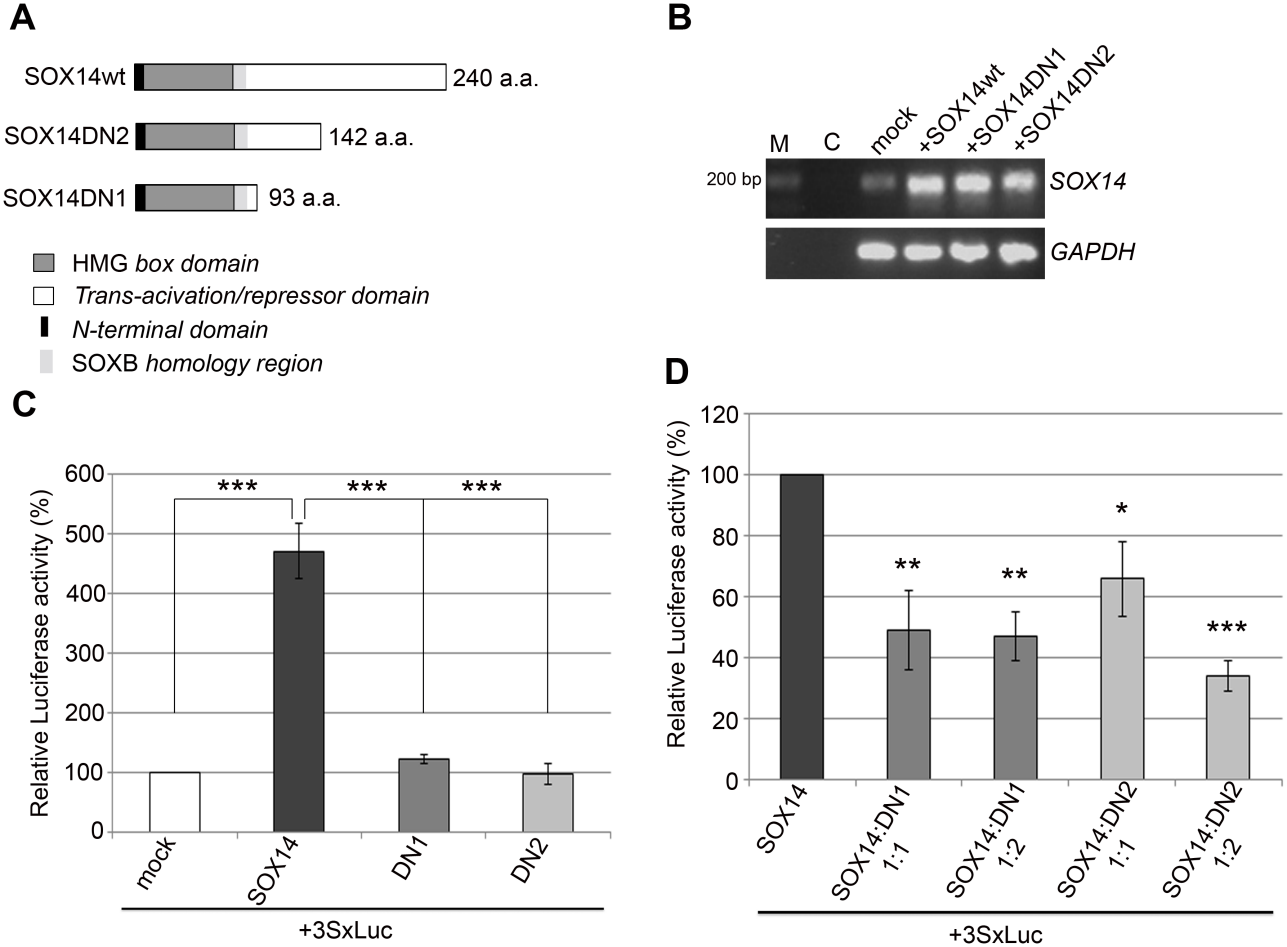
Generation and analysis of dominant negative forms of SOX14. **A**- Schematic representation of human SOX14wt protein and two truncated forms of SOX14 protein, lacking the C-terminal domain, SOX14DN2 (142 amino acids in length) and SOX14DN1 (93 amino acids in length). **B**—PCR analysis of *SOX14* expression upon transient transfection with empty vector (mock) and vectors expressing SOX14wt or truncated SOX14 protein (SOX14DN1 and SOX14DN2), as indicated. *GAPDH* was used as the loading control. Three independent experiments were performed, and one representative PCR is shown. M- DNA ladder, C- PCR negative control. **C**–Functional analysis of dominant negative SOX14 forms, a SOX responsive luciferase reporter plasmid 3xSXluc was transfected into HeLa cells in combination with either empty vector—pcDNA3.1 (mock), pcDNA3.1SOX14 (SOX14), pcDNA3.1SOX14DN1 (DN1) or pcDNA3.1SOX14DN2 (DN2). Normalized luciferase activities were calculated relative to the 3xSXluc activity in cells co-transfected with empty vector, which was set as 100% and relative to the 3xSXluc activity in cells co-transfected with SOX14. Data are presented as the means ± SEM of at least three independent experiments. Mean values of relative luciferase activities were compared with Student’s *t*-test and P-values calculated *** *p* ≤ 0.001. **D**- Competition assay. Increasing amounts of DN1 or DN2 expression vectors (1: 1 and 1: 2) were co-transfected with a fixed amount of SOX14wt expression vector and the luciferase reporter plasmid 3xSXluc. Normalized luciferase activities were calculated as percentages of the 3xSXluc activity in cells co-transfected with SOX14, which was set as 100%. Data are presented as the means ± SEM of at least three independent experiments. Mean values of relative luciferase activities were compared with Student’s *t*-test and P-values calculated *p ≤ 0.05, **p ≤ 0.01, ***p ≤ 0.001.

In order to evaluate the role of SOX14 in basic cellular processes we used transient overexpression of SOX14wt protein. Having in mind the close homology among SOX family proteins and previously described functional redundancy [18,19], studying the role of any SOX protein function using a knockdown strategy might be challenging. Instead, use of a dominant-negative mutant is a more promising approach. The major advantage of a dominant-negative mutant is its ability to block the binding site for the wild type protein which could consequently lead to inhibition of the effect of both SOX14wt and related SOX proteins able to bind the same target sequence. For that purpose we generated two truncated forms of SOX14 protein consisting of 93 (SOX14DN1) and 142 (SOX14DN2) amino acids in length (Fig 2A). Both truncated forms of SOX14 retained an intact HMG DNA binding domain and the SOXB homology region (Fig 2A) [20], while their trans-activation/repression domain was removed. Ectopic expression of SOX14wt and the truncated forms was confirmed by RT-PCR (Fig 2B).

In our previous study we showed that SOX14wt acts as a transcriptional activator of SOX-responsive luciferase reporter gene in HeLa cells [6]. We have used the same experimental approach in order to analyze functional properties of SOX14DN1 and SOX14DN2. Co-transfection experiments of 3xSXluc vector with empty pcDNA3.1, SOX14wt or dominant negative constructs revealed decreased transactivation ability of the truncated proteins compared to SOX14wt in HeLa cells (Fig 2C). In particular, while SOX14wt led to approximately 5-fold activation of reporter gene activity, dominant negative mutants exhibited low or no trans-activation activity.

The dominant negative effect of SOX14DN1 and SOX14DN2 was further tested in competition experiments where an increasing amount of either SOX14DN1 or SOX14DN2 expression constructs was co-transfected with a fixed amount of SOX14wt (Fig 2D). These data confirmed that both truncated forms of SOX14 protein were able to compete successfully for the binding site with SOX14wt protein. Besides its HMG box domain, each SOX protein has one or two other functional domains with transactivation, transrepression, or homodimerization features associated with control of target gene transcription [21,22]. By functional testing of SOX14 dominant negative forms, we identified that the region between amino acids 142 to 240, deleted within SOX14DN2, is crucial for full SOX14wt transactivation activity. Since both constructs showed similar dominant negative activity, construct SOX14DN1, which lacks most of the SOX14wt protein, was selected for further experiments.

### SOX14 overexpression had no effect on migration and invasion of HeLa cells

**Fig 3:**
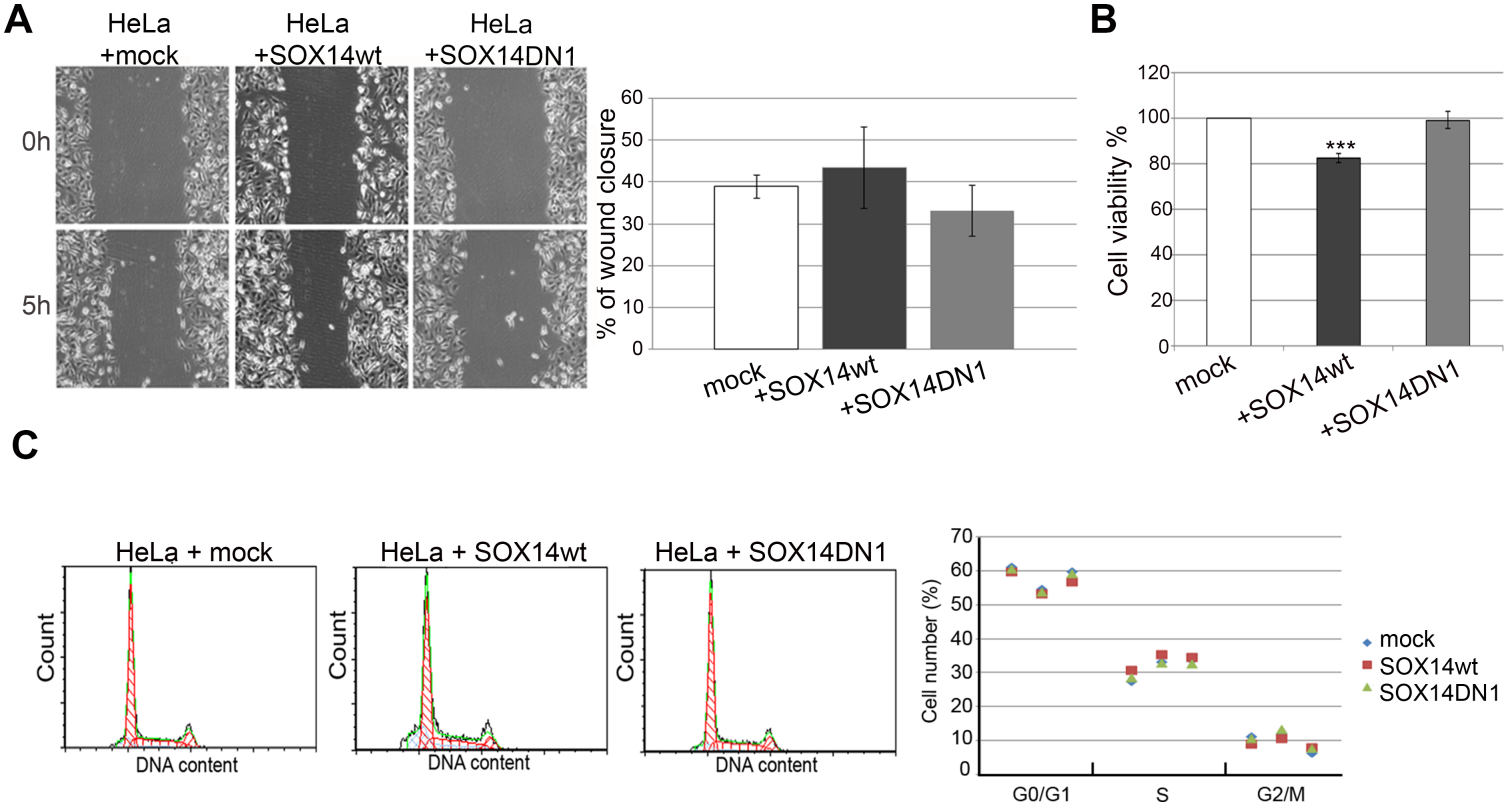
Assessment of the role of SOX14 in HeLa cell migration, viability and cell cycle. **A**- The effect of wt or DN1 SOX14 overexpression on migration of HeLa cells, wound-scratch migration assay. Representative images of cell migration are presented. The graph quantifies migration of the transfected cells 5 h after scratching. The changes in migration distance were quantified by measuring the difference in gap closure where gap width at 0 h was set as 100%. The results are presented as the means ± SEM of at least three independent experiments. **B**—MTS viability assay performed 24 h after transient transfection of HeLa cells with SOX14wt or SOX14DN1. Relative cell viability was calculated as a percentage of mock transfected HeLa cell viability that was set as 100%. Results are presented as the means ± SEM of at least three independent experiments. P-values were calculated using Student’s t-test, ***p≤0.001. **C**—Flow cytometry analysis of cell cycle distribution in HeLa cells 24 h after transfection with empty vector (mock), SOX14wt or SOX14DN1. One representative analysis is shown in the left panel, while the results of three independent experiments for cell cycle distribution are presented on dot plot.

It is widely known that tumor progression is associated with cell migration, invasion and metastasis. Our aim was to assess the effect of SOX14 protein on migration and invasion of HeLa cells. We used wound scratch healing methodology to examine the effect of SOX14wt and SOX14DN1 overexpression on the ability of HeLa cells to close the wounded area. No significant changes in cell motility were observed upon transient overexpression of both wild type and dominant negative SOX14 proteins in HeLa cells (Fig 3A). These results indicated that neither SOX14wt nor its dominant negative counterpart affected the migration potential of HeLa cells in the wound scratch assay. In addition, we performed the Transwell migration/invasion assay in order to elucidate whether overexpression of SOX14wt is able to affect cell migration through an uncoated membrane or a membrane coated with extracellular matrix proteins. As expected, SOX14wt had no effect on cell migration or invasion through extracellular matrix (S1A and S1B Fig). Considering these results, we concluded that SOX14 is not involved in the regulation of migration of Hela cells.

### SOX14 overexpression reduces viability and promotes apoptosis in HeLa cells

We then analyzed whether SOX14wt overexpression affects proliferation and apoptosis in HeLa cells. By MTS assay, we observed that SOX14wt overexpression decreased cell viability by approximately 20% (Fig 3B), while SOX14DN1 gave a similar percentage of viable cells as mock transfection (Fig 3B).

Next, we tested whether SOX14 ectopic expression could affect cell cycle distribution. Twenty-four hours after transfection with SOX14wt, flow cytometry analysis revealed a slight increase of cell percentage in S phase of the cell cycle, together with a decrease in the G2/M phase (Fig 3C). These results suggest that SOX14 overexpression induced minor accumulation of cells in S phase of the cell cycle.

**Fig 4:**
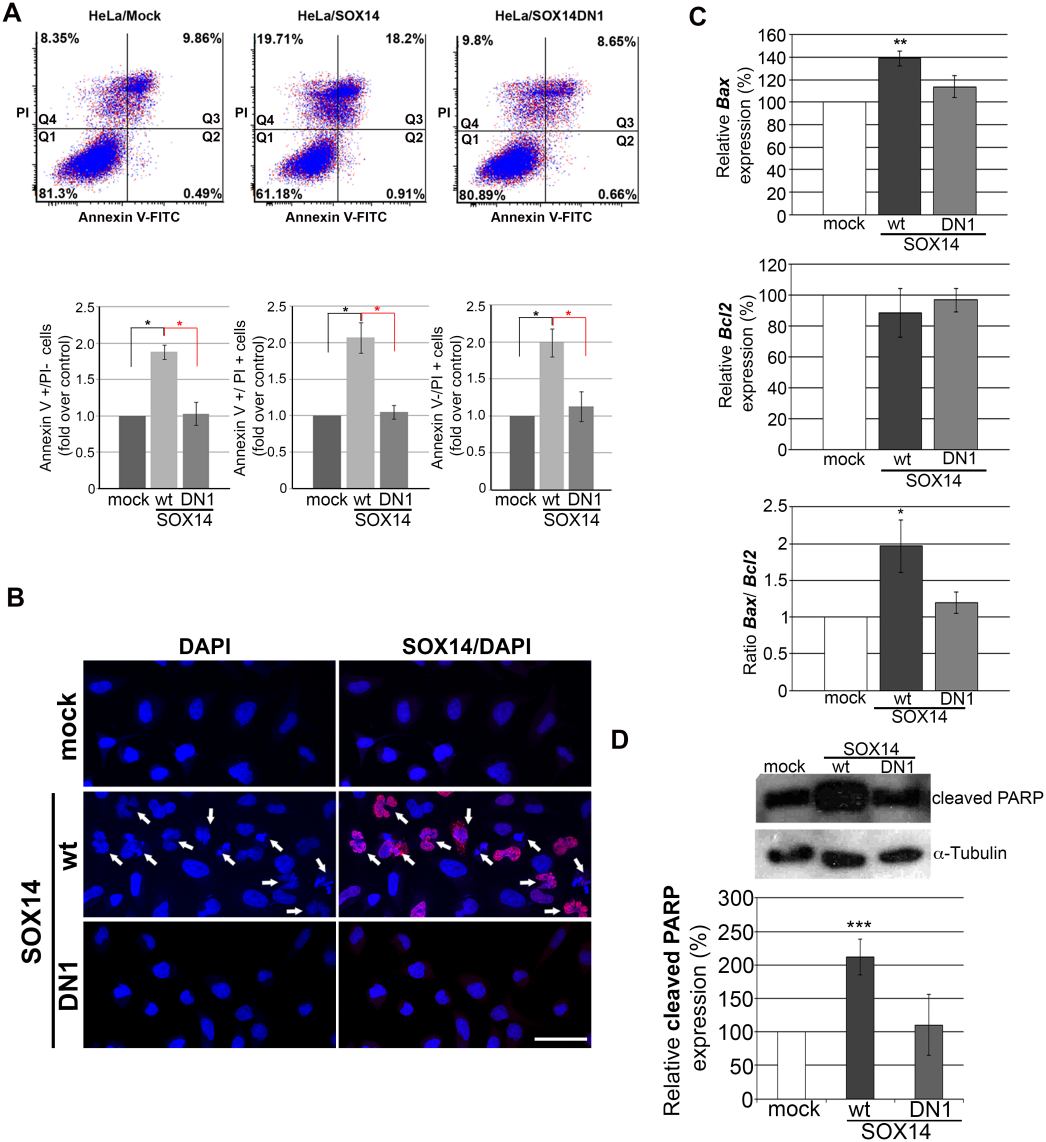
Assessment of the role of SOX14 in HeLa cell apoptosis. **A**. Flow cytometry analysis of Annexin-FITC staining and propidium iodide (PI) accumulation in HeLa cells 24 h after transfection with empty vector (mock), SOX14wt or SOX14DN1. Upper panel: One representative analysis is presented in each quadrant. Q1: PI-/Annexin- cells (live cells); Q2: PI-/Annexin V+ cells (early apoptosis); Q3: PI+/Annexin+ cells (late apoptosis); Q4: PI+/Annexin- cells (necrotic cells). Lower panel: Quantitative analyses of PI and Annexin V positive cells are shown on the histograms relative to the control (mock), which was set as 1, as means ± SEM of three independent experiments. Mean values were compared with Student's *t*-test and P-values calculated, *p ≤ 0.05. **B**- DAPI staining of HeLa cell nuclei after mock, SOX14wt or SOX14DN1 transient transfection. Cells with fragmented nuclei are indicated by arrows. **C**- qRT-PCR analysis of *Bax* and *Bcl*2 gene expression after transient transfection with empty vector (mock) and vectors expressing SOX14wt or truncated SOX14 protein (SOX14DN1), as indicated. GAPDH was used as the loading control. The effect of SOX14 overexpression on *Bax* and *Bcl*2 genes is shown in graphs. The quantities of *Bax* and *Bcl2* genes in transfected cells were calculated as percentages of their respective expression levels in mock transfected cells, which were set as 100%. The Bax/Bcl-2 ratio was calculated from their relative mRNA expression. Data are presented as the means ± SEM of three independent transfection experiments. Mean values were compared with Student's t-test and P-values calculated *p ≤ 0.05,**p ≤ 0.01 **D**—Western blot analysis of cleaved PARP expression in HeLa cells transfected with either empty vector (mock), SOX14wt or DN1 expression construct for 24 h. α- Tubulin was used as the loading control. The effect of SOX14 overexpression on cleaved PARP protein level is presented in the graph. The quantities of cleaved PARP protein in transfected cells were calculated as a percentage of cleaved PARP in mock transfected cells which was set as 100%. Data are shown as the means ± SEM of three independent transfection experiments. Mean values were compared with Student's t-test and P-values calculated, ***p ≤ 0.001.

Annexin V/propidium iodide double staining analysis revealed that SOX14wt overexpression led to an increase in cell death (Fig 4A). The number of both apoptotic (early apopototic- Annexin V+ and late apoptotic- Annexin V+/PI+) and necrotic cells (PI+) was raised approximately 2-fold, 24 h after SOX14 transfection (Fig 4A). SOX14DN1 failed to activate apoptosis and the number of cells in each quadrant was similar to mock transfection (Fig 4A). In addition, following DAPI staining, we observed more cells with misshaped or fragmented nuclei in cultures transfected with SOX14wt compared to mock or SOX14DN1 treatment (Fig 4B).

To further examine SOX14 involvement in apoptosis, we analyzed the effect of SOX14 on expression of several apoptosis related markers. The results of qRT-PCR showed increased expression of the proapoptotic *Bax* gene and no changes in anti-apoptotic *Bcl2* gene expression 24 h after transfection with SOX14wt (Fig 4C). At the same time SOX14DN1 had no effect on either *Bax* or *Bcl2* gene expression (Fig 4C). These findings imply that overexpression of SOX14 enhances the ratio of Bax/Bcl-2 at the transcriptional level (Fig 4C), leading to apoptosis in HeLa cells. An additional hallmark of apoptosis, cleaved-PARP, was analyzed by Western blot, which revealed that SOX14wt increased cleaved-PARP level in HeLa cells approximately 2- fold, while SOX14DN1 had no effect (Fig 4D).

The presented results indicate that SOX14 initiated the process of apoptosis in HeLa cells as revealed by Annexin V/PI staining, increased expression of the pro-apoptotic *Bax* gene and cleaved PARP.

### SOX14 overexpression increased p53 protein in HeLa cells

**Fig 5:**
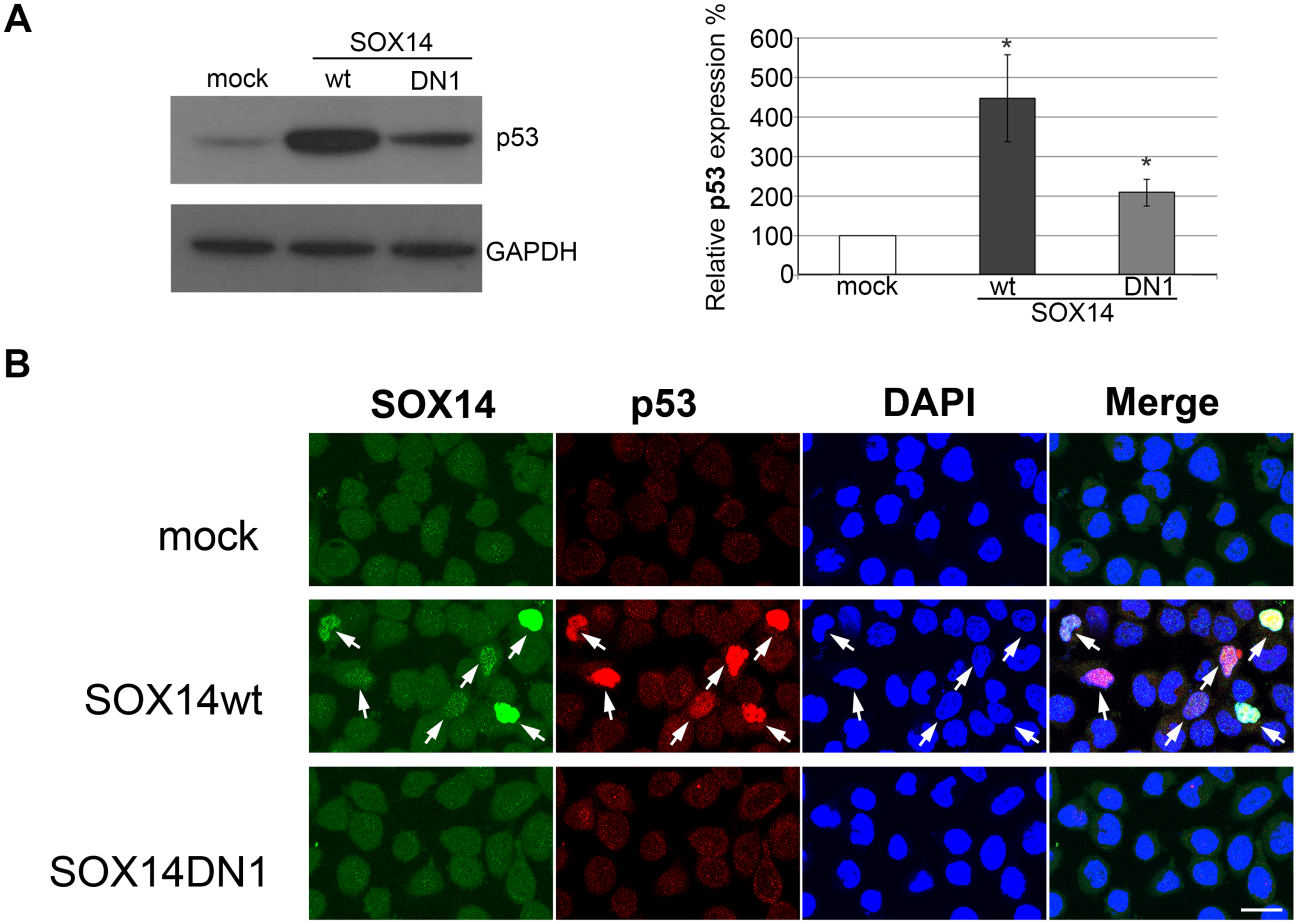
The effect of SOX14wt and SOX14DN1 on p53 protein in HeLa cells. **A**- Western blot analysis of p53 protein expression in HeLa cells transfected with either empty vector (mock), SOX14wt or SOX14DN1. GAPDH was used as the loading control. Quantification of the effect of SOX14 overexpression on p53 protein level is presented in the graph. Amounts of p53 protein in transfected cells were calculated as a percentage of the p53 in mock transfected cells, which was set as 100%. Data are given as the means ± SEM of three independent transfection experiments. Mean values were compared with Student's t-test and. P- values calculated, *p ≤ 0.05. **B**—The effect of SOX14 overexpression on p53 protein level detected by immunocytochemistry. Cells were transfected with empty vector (mock), SOX14wt or SOX14DN1. Cell nuclei were counterstained with DAPI. Scale bar: 20 μm.

Since SOX14wt overexpression induced apoptosis in HeLa cells, we analyzed whether this activity could be mediated through p53 signaling. First, we tested whether p53 protein is affected by SOX14 overexpression. By Western blot we revealed significant accumulation of p53 protein in HeLa cells 24 h upon transfection with SOX14wt, while the dominant negative mutant of SOX14 did not induce this level of p53 accumulation (Fig 5A). The effect of SOX14wt overexpression on p53 protein level was also confirmed immunocytochemically (Fig 5B).

### SOX14 overexpression induced stabilization of p53

**Fig 6:**
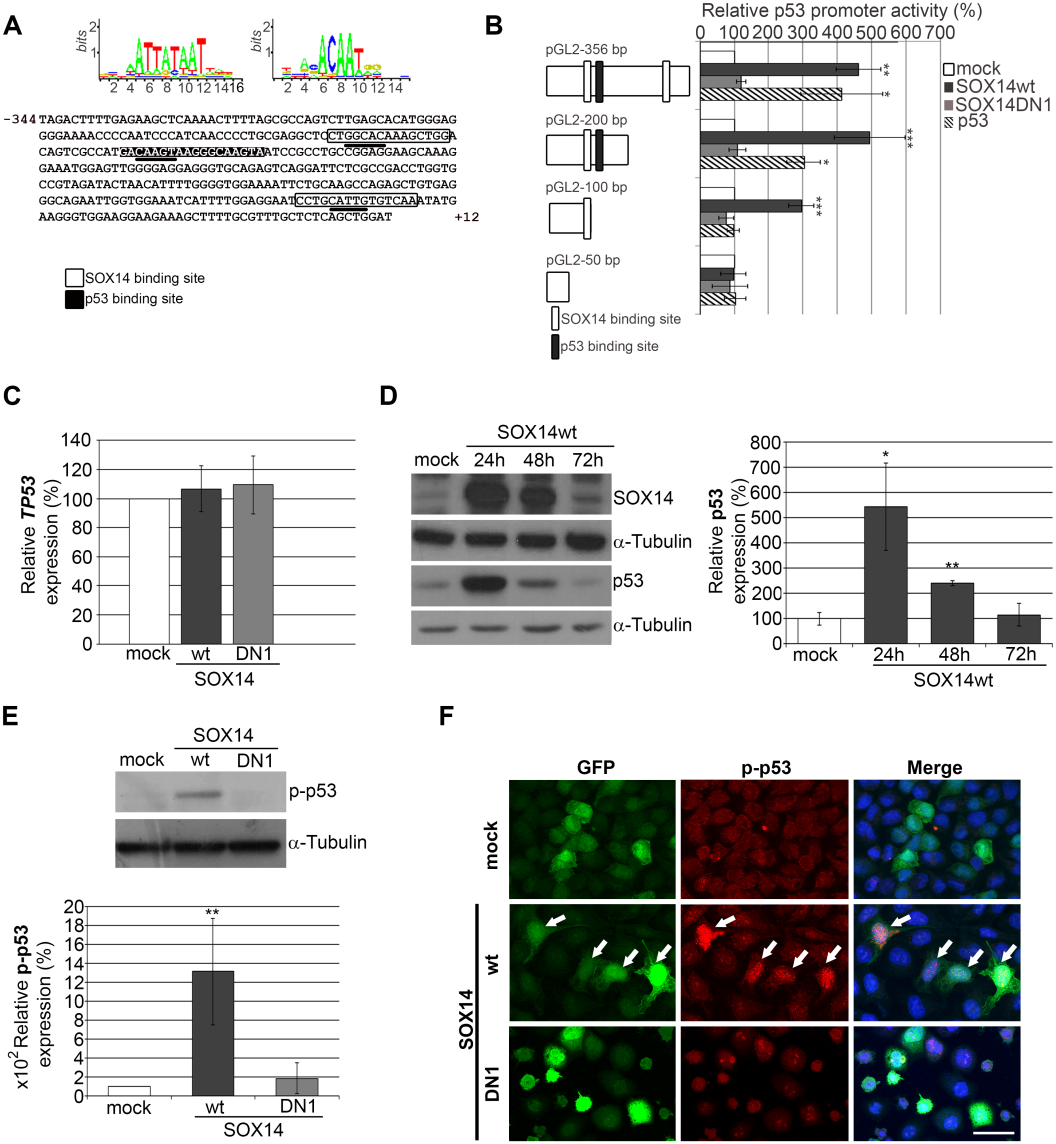
The effect of SOX14wt overexpression on p53 transcription and stability in HeLa cells. **A**-The *TP53* promoter sequence (fragment −344 to +12 relative to the first transcription initiation site) with labeled consensus binding sites for SOX14 (white) and p53 (black); core sequences are underlined. **B**- Luciferase assay and schematic representation for *TP53* luciferase promoter constructs. Position of SOX14 (white) and p53 (black) binding sites are indicated. Each of the represented *TP53* promoter constructs was transfected in combination with either empty vector (mock), SOX14wt, SOX14DN1 or p53 expression vector. Normalized luciferase activities were calculated in relation to the adequate *TP53* luciferase promoter vector activity in cells co-transfected with empty vector, which was set as 100%. Data are presented as the means ± SEM of at least three independent experiments. Mean values of relative luciferase activities were compared with Student’s t-test. P-values are *p≤ 0.05, **p≤0.01 and ***p≤0.001. **C-** qRT-PCR analysis of *TP53* gene expression upon transient transfection with empty vector (mock) and vectors expressing SOX14wt or truncated SOX14 protein (SOX14DN1), as indicated. *GAPDH* was used as the loading control. The effect of SOX14 overexpression on *TP53* gene level is presented in the graph. *TP53* gene expression in transfected cells is calculated as a percentage of the amount in mock transfected cells, which was set as 100%. Data are presented as the means ± SEM of three independent transfection experiments. Mean values were compared with Student's t-test. **D**- Western blot analysis of p53 expression in HeLa cells transfected with either empty vector (mock) or the SOX14wt expression construct after 24 h, 48 h and 78 h. α- Tubulin was used the loading control. The effect of SOX14 overexpression on p53 protein level is shown in the graph. Quantities of p53 protein in transfected cells were calculated as a percentage of that in mock transfected cells, which was set as 100%. Data are shown as the means ± SEM of three independent transfection experiments. Mean values were compared with Student's t-test and P-values calculated, *p ≤ 0.05, **p ≤ 0.01. **E**- Western blot analysis of phospho-p53 (p-p53) expression in HeLa cells transfected with either empty vector (mock), SOX14wt or SOX14DN1. α-Tubulin was used as the loading control. The graph shows quantification of the effect of SOX14 overexpression on phospho-p53 protein level in transfected cells calculated as a percentage of the phospho-p53 in mock transfected cells, which was set as 100%. Data are presented as the means ± SEM of three independent transfection experiments. Mean values were compared with Student's t-test and P-values calculated, **p ≤ 0.01. **F**-The effect of SOX14 overexpression on phospho-p53 protein level detected immunocytochemically. Cells were co-transfected with pEGFP-C1 and empty vector (mock), SOX14wt or SOX14DN1. Cell nuclei were counterstained with DAPI. Arrows indicate phospho-p53 immunoreactivity in transfected cells. Scale bar: 20 μm.

In order to assess whether SOX14 activates p53 transcriptionally we analyzed putative transcription factor binding sites within the *TP53* promoter (GenBank: J04238.1) *in silico* [23]. Using the Jaspar program (http://jaspar.genereg.net/) [24] we found two SOX14 binding motifs in the *TP53* promoter region (Fig 6A). In order to test whether SOX14 could regulate *TP53* promoter activity, we performed luciferase reporter gene assays using the *TP53* promoter, (pGL2-356bp), together with three p53 promoter deletion constructs (pGL2-200bp, pGL2-100bp and pGL2-50bp) (schematic representation in Fig 6B) [25]. We observed that SOX14wt significantly enhanced activity of the pGL2-356bp construct (encompassing the whole *TP53* promoter) (Fig 6B). Significant luciferase activity was also observed in co-transfection experiments with SOX14wt and pGL2-200bp or pGL2-100bp constructs (Fig 6B), while no luciferase activity was observed in co-transfection with the pGL2-50bp construct (Fig 6B) which lacks the SOX14 binding site. At the same time, SOX14DN1 protein exhibited no effect either on *TP53* promoter reporter activity, or on *TP53* promoter deletion constructs (Fig 6B).

It is well known that p53 regulates its own expression [26] and for that reason we used the p53wt expression construct as a positive control. The obtained results showed that p53 overexpression increased luciferase activity of constructs bearing the p53 binding site (pGL2-356bp and pGL2-200bp constructs) (Fig 6B), while no luciferase activity was observed for co-transfection with constructs lacking the p53 binding site (pGL2-100bp and pGL2-50bp) (Fig 6B).

These experiments showed that SOX14 was able to activate *TP53* promoter reporter constructs in a manner that correlates with the number and position of putative SOX14 binding sites, as was shown for p53 itself.

Furthermore, we analyzed *TP53* expression upon transient transfection of SOX14wt or SOX14DN1. Surprisingly, qRT-PCR evaluation of *TP53* expression revealed that *TP53* gene expression did not change (Fig 6C). It is well known that p53 is an unstable protein, the amount of which is known to be regulated by the Mouse double minute 2 homolog-Mdm-2–ubiquitin—proteasome degradation pathway [16]. We determined the level of p53 upon transfection of SOX14wt for 72 h and revealed significant p53 accumulation up to 48 h. Overexpression of SOX14wt induced the expression of p53 protein in HeLa cells, approximately 4-fold, 24 h after transfection (Fig 6D). At 48 h after transfection, the expression of p53 was still increased approximately 2- fold when compared to mock transfected HeLa cells, while 72 h after transfection expression of p53 was at the basal level (Fig 6D). This finding argues that SOX14 could contribute to p53 stability (Fig 6D).

It is widely known that phosphorylation of serine at position 15 is one of the mechanisms for p53 stabilization [27,28]. Therefore, we performed Western blot analysis with a specific phospho-p53 (Ser15) antibody and showed that overexpression of SOX14wt increased phospho- p53 protein level approximately 10-fold, 24 h after transfection (Fig 6E). On the other hand, the dominant negative mutant of SOX14 had no effect on phospho-p53 protein 24 h after transfection (Fig 6E). This effect of SOX14wt overexpression on phospho-p53 Ser15 was also confirmed immunocytochemically (Fig 6F). The findings indicated that, although SOX14 was able to induce p53 promoter activity, no effect on transcription was detected. Instead, we have shown that SOX14 overexpression contributed to stabilization of p53 protein in HeLa cells by increasing the amount of phosphorylated p53 at position Ser15.

### SOX14 overexpression transactivates *CDKN1A*/p21^Waf1/Cip1^ promoter and increases the expression of *CDKN1A/* p21 ^Waf1/Cip1^ in HeLa cells

**Fig 7:**
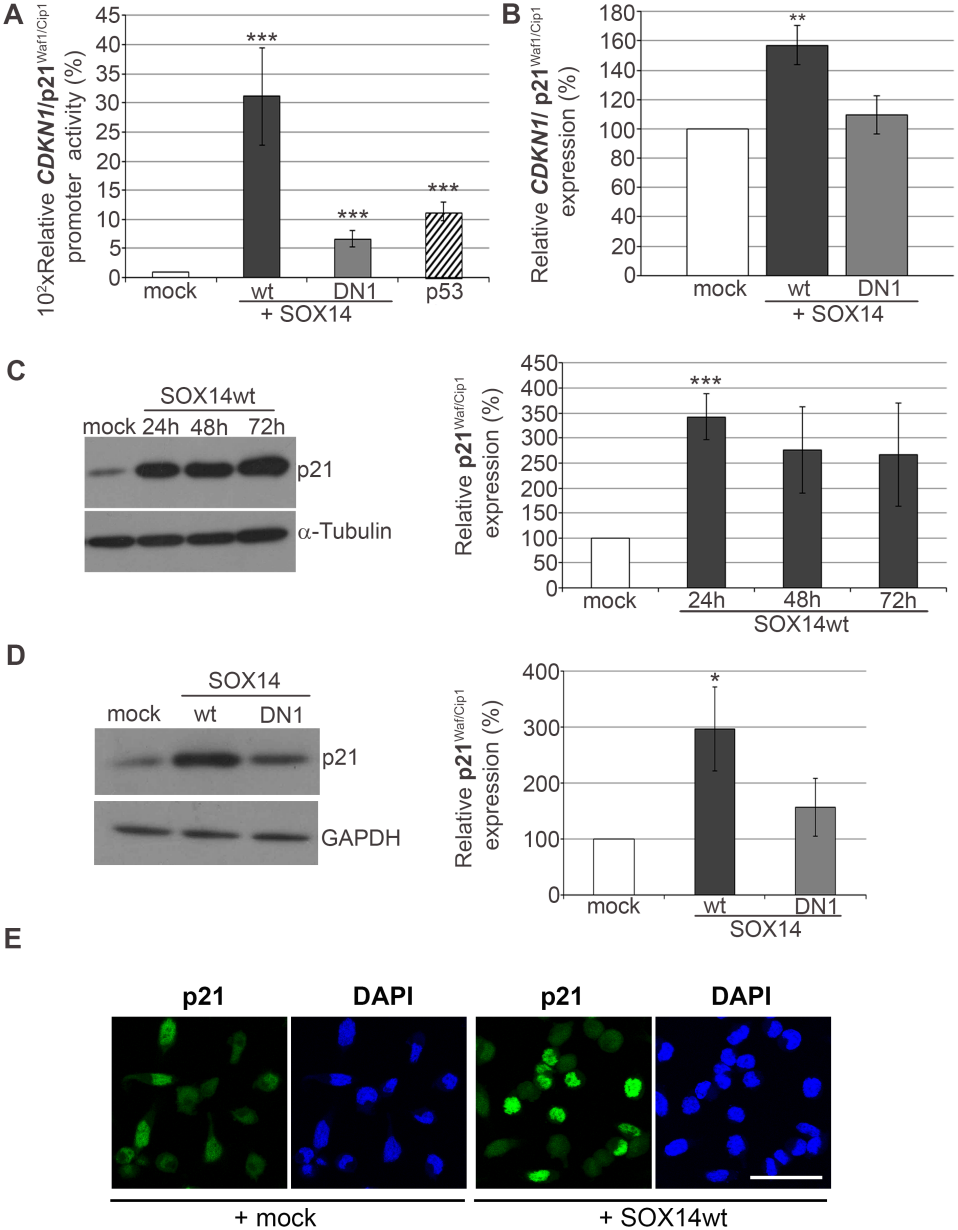
The effect of SOX14 ectopic expression on p21^Waf1/Cip1^ expression in HeLa cells. **A**- Effect of SOX14 ectopic expression on activity of the *CDKN1A*/p21^Waf1/Cip1^ promoter. The plasmid p21-Luc was co-transfected into HeLa cells with pcDNA3.1 vector, SOX14wt, SOX14DN1 or the p53 expression construct. Normalized luciferase activities were calculated relative to the p21-Luc activity in cells co-transfected with pcDNA3.1 vector, which was set as 100%. Data are presented as the mean ± SEM of at least three independent transfections. Mean values of relative luciferase activities were compared with Student's t-test and P-values are ***p ≤ 0.001. **B**- qRT-PCR analysis of *CDKN1A*/p21^Waf1/Cip1^ gene expression upon transient transfection with empty vector (mock) and vectors expressing SOX14wt or truncated SOX14 protein (SOX14DN1), as indicated. *GAPDH* was used as the loading control. Quantification of the effect of SOX14 overexpression on *CDKN1A*/p21^Waf1/Cip1^ gene level is presented in the graph. *CDKN1A*/p21^Waf1/Cip1^ gene expression in transfected cells was calculated as a percentage of the expression level in mock transfected cells, which was set as 100%. Data are given as the means ± SEM of three independent transfection experiments. Mean values were compared with Student's t-test. **p ≤ 0.01. **C**—Western blot analysis of p21^Waf1/Cip1^ expression in HeLa cells transfected with either pcDNA3.1 vector (mock) or SOX14wt expression construct, for 24 h, 48 h and 78 h. α- Tubulin was used as the loading control. The effect of SOX14 overexpression on p21^Waf1/Cip1^ protein level is presented in graphs. The quantities of p21^Waf1/Cip1^ protein in transfected cells were calculated reltive to the amount in cells transfected with pcDNA3.1 vector, which was set as 100%. Data are presented as the means ± SEM of three independent transfection experiments. Mean values were compared with Student's t-test and P-values were calculated, ***p ≤ 0.001. **D**—Western blot analysis of p21^Waf1/Cip1^ expression in HeLa cells transfected with either pcDNA3.1 vector (mock), SOX14wt or SOX14DN1. GAPDH was used as the loading control. The quantitative effect of SOX14 overexpression on p21 ^Waf1/Cip1^ protein level is presented in the graph. Amounts of p21 ^Waf1/Cip1^ protein in transfected cells were calculated as a percentage of that in cells transfected with pcDNA3.1 vector, which was set as 100%. Data are presented as the means ± SEM of three independent transfection experiments. Mean values were compared with Student's t-test and P-values calculated, *p ≤ 0.05. **E**—The effect of SOX14 overexpression on p21^Waf1/Cip1^ protein level detected by immunocytochemistry. Cells were transfected with empty vector (mock) or SOX14wt. Cell nuclei were counterstained with DAPI. Scale bar: 50 μm.

In order to test the functional relevance of SOX14 induced p53 accumulation, we analyzed the effect of SOX14 overexpression on the well-known p53 target gene *CDKN1A*/p21^Waf1/Cip1^. This gene encodes an inhibitor of cyclin-dependent kinases that control cell cycle progression and negatively regulate cellular proliferation [29–34]. For this purpose we used a promoter luciferase reporter construct of *CDKN1A*/p21^Waf1/Cip1^ (p21-Luc) in transient co-transfection experiments with pcDNA3.1, SOX14wt or SOX14DN1. Luciferase activities were measured 24 h after transfection and the obtained results showed that SOX14 transactivated *CDKN1A*/p21^Waf1/Cip1^ promoter activity approximately 30-fold (Fig 7A). In contrast, the truncated form of SOX14 did not stimulate this level of *CDKN1A*/p21^Waf1/Cip1^ promoter activation (Fig 7A). These results showed that SOX14 could induce promoter activity of the p53 target gene, *CDKN1A*/p21^Waf1/Cip1^. Interestingly, this promoter gave greater activation by SOX14 overexpression than did p53 (Fig 7A). These results open the possibility that *CDKN1A*/p21^Waf1/Cip1^ could be a direct target of the SOX14 transcription factor and that SOX14 and p53 display synergism in regulation of *CDKN1A*/p21^Waf1/Cip1^ expression.

Furthermore, by qRT-PCR we have shown that expression of *CDKN1A*/p21^Waf1/Cip1^ was increased at the mRNA level 24 h after transfection with SOX14wt in comparison with the mock control (Fig 7B). The Western blot revealed that 24 h after transfection, SOX14wt also increased p21^Waf1/Cip1^ protein to a level 3x higher than with mock transfection (Fig 7C). The level of p21^Waf1/Cip1^ protein remained elevated up to 72 h after transfection of SOX14wt (Fig 7C), probably due to p53 protein stabilization after SOX14wt overexpression. At the same time, 24 h upon transfection, SOX14DN1 did not induce p21^Waf1/Cip1^ expression to the same extent (Fig 7D). Upregulation of p21^Waf1/Cip1^ expression upon SOX14wt overexpression was also confirmed by ICC (Fig 7E). These results indicate that SOX14-induced p53 stabilization leads to activation of p21^Waf1/Cip1^ expression, but we cannot exclude the possibility that *CDKN1A*/p21^Waf1/Cip1^ could also be a direct target of the SOX14 transcription factor.

## Discussion

For many years the *SOX14* gene was one of the least studied *SOX* genes, with a particular lack of functional investigations regarding its role in cancerogenesis. Current literature data are unclear whether this transcription factor acts as a tumor suppressor or oncogene in cervical carcinoma. This paper presents a comprehensive analysis of the role of SOX14 in a variety of cellular processes in cells derived from cervical adenocarcinoma. Our results shed light on its activity in regulating programmed cell death, for the first time pointing to the influence of SOX14 on stabilization of the well known genome keeper p53.

For more than a decade SOX14 was considered to be a transcriptional repressor [20]. Here we have revealed that SOX14 protein has transactivation properties on both *TP53*/p53 and *CDKN1A/* p21^Waf1/Cip1^ promoter constructs, which supports our previous suggestion that the strict division of SOXB proteins into activators and repressors needs to be reevaluated [6]. Its methylation status in HeLa cells correlates with results obtained for cervical tumor samples from patients [10,11]. These showed that, SOX14 cannot exert its potential tumor-suppressive role due to hypermethylation. However, when overexpressed it triggers the accumulation of p53 in HeLa cell nuclei, indicating a potential to overcome p53 degradation.

The p53 tumor suppressor is one of the most important proteins for preventing cancer [35–37]. Wild type p53 is a key molecule that controls cell proliferation through cell cycle arrest or apoptosis in response to a variety of stressors. Loss of these regulatory functions is a major event contributing to genomic instability, transformation and malignancy [21,38]. The importance of *TP53* in tumor suppression is reflected by the fact that it is the most frequently mutated gene identified in human malignancies [39,40]. Therefore, discovery of genes involved in the regulation of p53 is crucial for further understanding of mechanisms critical for cancerogenesis. This is the first time that SOX14 is shown to be involved in up-regulation of the p53 signaling pathway, as demonstrated by stabilization of p53, which suggests some tumor suppressor activity in this cervical cancer cell line. Although SOX14 overexpression increased activity of the *TP53* promoter, surprisingly no change in the level of the *TP53* transcript was observed. This led us to assume that SOX14 could contribute to p53 stabilization. Further analysis unambiguously showed that SOX14 significantly increased the amount of phospho-p53 (Ser-15) in HeLa cells confirming that SOX14 triggers posttranslational modification of p53, necessary for stabilization.

It is well known that HeLa cells express the E6 oncoprotein of *Human papilloma virus 18* (HPV-18). This causes ubiquitin mediated degradation and inhibition of phosphorylation of wild type p53 protein and thus makes HeLa cells p53 deficient [41,42]. We observed that p53 accumulation correlated with the level of SOX14 overexpression, with the highest level at 24 h upon transfection and continuous elevation for up to 48 h. Previous findings suggest that the half-life of ectopically expressed p53 in HeLa cells is approximately 3 hours [43], which was presumed to be the result of the E6-activated p53-degradation activity present in these cells. Our results demonstrated that SOX14 induced p53 accumulation apparently overrides E6-mediated p53 degradation. However, further work is needed to elucidate whether SOX14 participates directly in the process of phosphorylation, or controls expression of genes that regulate phosphorylation or somehow participates in deactivation of E6 protein.

Our analysis showed that p53 target genes, cyclin-dependent kinase inhibitor p21 ^Waf/Cip1^ [30] and proapototic gene *Bax* [44] were upregulated upon SOX14 overexpression, leading us to strengthen our conclusion about the involvement of SOX14 in activation of the p53 signaling pathway. It is widely known that p53-dependent cycle arrest is primarily mediated by p21 ^Waf/Cip1^ [30]. Although SOX14 significantly elevated expression of p21 ^Waf/Cip1^, it had only a slight effect on cell cycle distribution. Further work is needed to elucidate whether SOX14 has a more prominent role in cell cycle regulation. On the other hand, the effect of SOX14 on apoptosis was more evident. The presented results indicate that the effect of SOX14 on cellular processes, particularly apoptosis, is probably dependent on p53 signaling activation. Restitution of wild type p53 function in HeLa cells mediated by SOX14 could provide a basis for a novel therapeutic approach which could be applied to overcome the malignant phenotype.

It is already known that several members of the *SOX* family affect p53 activity, enabling it to act as a tumor suppressor in different types of cancer [45–47]. SOX4 was demonstrated to interact with and stabilize p53 protein by blocking Mdm2- mediated p53 ubiquitination and degradation. Also, it enhances p53 acetylation by interacting with p300/CBP and facilitating p300/CBP/p53 complex formation, which induces apoptosis and cell cycle arrest *in vitro* [48]. On the other hand, in hepatocellular carcinoma cell lines SOX4 interacts with p53 and this association in turn modulates p53-mediated transcription at the *Bax* promoter, leading to inhibition of apoptosis via suppression of *Bax* gene expression [46]. Moreover, it was shown that SOX30 is an epigenetically silenced tumor suppressor, which promotes tumor cell apoptosis by transcriptional activation of p53 in lung cancer [45]. Our results have added SOX14 to the list of SOX family members that can exert a tumor suppressor role, by increasing p53 stability in cervical carcinoma cells *in vitro*.

## Conclusions

In this paper, we have shown that the *SOX14* promoter is methylated in a cervical cancer cell line. By functional testing of SOX14 dominant negative forms, we have identified the region crucial for full SOX14 transactivation activity. Using the overexpression and dominant negative approach we have concluded that SOX14 overexpression reduces cell viability and increases the number of cells undergoing apoptosis. In addition, we have demonstrated that SOX14 increased the stable, active, phosphorylated form of p53 in cervical cancer cells *in vitro*, confirming that SOX14 can trigger posttranslational modification of p53. Furthermore, SOX14 induced upregulation of *CDKN1A*/p21^Waf1/Cip1^ expression, which is presumably mediated by activated p53 protein. The obtained results have revealed for the first time, that tumor suppressor activity by SOX14 *in vitro* is mediated by activation of the p53 signaling pathway.

## Supporting information

S1 Fig Assessment of the role of SOX14 in HeLa cell migration and invasion. **A**—Transwell migration assay on HeLa cells transfected with empty vector (mock) or SOX14wt. Representative images of the transwell migration assay are presented. The relative change in cell migration was calculated as a percentage of HeLa cell migration after mock transfection that was set as 100%. Cells were counted from five fields and averages were calculated. Results are presented as the means ± SEM of at least three independent experiments performed in duplicate. **B**—Transwell invasion assay on HeLa cells transfected with empty vector (mock) or SOX14wt. Representative images of the transwell invasion assay are presented. The relative change in cell invasion was calculated as a percentage of HeLa cell invasion after mock transfection that was set as 100%. Cells were counted from five fields and averages were calculated. Results are presented as the means ± SEM of at least three independent experiments performed in duplicate.
(TIF)

## References

[1] Dong C, Wilhelm D, Koopman P (2004) Sox genes and cancer. Cytogenet Genome Res 105: 442–447. doi: 10.1159/000078217 15237232 10.1159/000078217

[2] Prior HM, Walter MA (1996) SOX genes: architects of development. Mol Med 2: 405–412. 8827711 PMC2230175

[3] Kiefer JC (2007) Back to basics: Sox genes. Dev Dyn 236: 2356–2366. doi: 10.1002/dvdy.21218 17584862 10.1002/dvdy.21218

[4] Zhu Y, Li Y, Jun Wei JW, Liu X (2012) The role of Sox genes in lung morphogenesis and cancer. Int J Mol Sci 13: 15767–15783. doi: 10.3390/ijms131215767 23443092 10.3390/ijms131215767 PMC3546660

[5] Hargrave M, Karunaratne A, Cox L, Wood S, Koopman P, Yamada T (2000) The HMG box transcription factor gene Sox14 marks a novel subset of ventral interneurons and is regulated by sonic hedgehog. Dev Biol 219: 142–153. doi: 10.1006/dbio.1999.9581 10677261 10.1006/dbio.1999.9581

[6] Popovic J, Stanisavljevic D, Schwirtlich M, Klajn A, Marjanovic J, Stevanovic M (2014) Expression analysis of SOX14 during retinoic acid induced neural differentiation of embryonal carcinoma cells and assessment of the effect of its ectopic expression on SOXB members in HeLa cells. PLoS One 9: e91852 doi: 10.1371/journal.pone.0091852 24637840 10.1371/journal.pone.0091852 PMC3956720

[7] Natarajan A, Yardimci GG, Sheffield NC, Crawford GE, Ohler U (2012) Predicting cell-type-specific gene expression from regions of open chromatin. Genome Res 22: 1711–1722. doi: 10.1101/gr.135129.111 22955983 10.1101/gr.135129.111 PMC3431488

[8] Tong WG, Wierda WG, Lin E, Kuang SQ, Bekele BN, Estrov Z, et al (2010) Genome-wide DNA methylation profiling of chronic lymphocytic leukemia allows identification of epigenetically repressed molecular pathways with clinical impact. Epigenetics 5: 499–508. doi: 10.4161/epi.5.6.12179 20484983 10.4161/epi.5.6.12179 PMC3322493

[9] Li F, Wang T, Tang S (2015) SOX14 promotes proliferation and invasion of cervical cancer cells through Wnt/beta-catenin pathway. Int J Clin Exp Pathol 8: 1698–1704. 25973056 PMC4396331

[10] Wang R, van Leeuwen RW, Boers A, Klip HG, de Meyer T, Steenbergen RD, et al (2016) Genome-wide methylome analysis using MethylCap-seq uncovers 4 hypermethylated markers with high sensitivity for both adeno- and squamous-cell cervical carcinoma. Oncotarget. 10.18632/oncotarget.12598 PMC5348351 27738327

[11] Senchenko VN, Kisseljova NP, Ivanova TA, Dmitriev AA, Krasnov GS, Kudryavtseva AV, et al (2013) Novel tumor suppressor candidates on chromosome 3 revealed by NotI-microarrays in cervical cancer. Epigenetics 8: 409–420. doi: 10.4161/epi.24233 23478628 10.4161/epi.24233 PMC3674050

[12] Andrews PW (1984) Retinoic acid induces neuronal differentiation of a cloned human embryonal carcinoma cell line in vitro. Dev Biol 103: 285–293. 6144603 10.1016/0012-1606(84)90316-6

[13] Arsic N, Rajic T, Stanojcic S, Goodfellow PN, Stevanovic M (1998) Characterisation and mapping of the human SOX14 gene. Cytogenet Cell Genet 83: 139–146. 9925951 10.1159/000015149

[14] Petrovic I, Milivojevic M, Popovic J, Schwirtlich M, Rankovic B, Stevanovic M (2015) SOX18 Is a Novel Target Gene of Hedgehog Signaling in Cervical Carcinoma Cell Lines. PLoS One 10: e0143591 doi: 10.1371/journal.pone.0143591 26588701 10.1371/journal.pone.0143591 PMC4654472

[15] Popovic J, Stevanovic M (2009) Remarkable evolutionary conservation of SOX14 orthologues. J Genet 88: 15–24. 19417540 10.1007/s12041-009-0003-4

[16] Haupt Y, Maya R, Kazaz A, Oren M (1997) Mdm2 promotes the rapid degradation of p53. Nature 387: 296–299. doi: 10.1038/387296a0 9153395 10.1038/387296a0

[17] Santi DV, Norment A, Garrett CE (1984) Covalent bond formation between a DNA-cytosine methyltransferase and DNA containing 5-azacytosine. Proc Natl Acad Sci U S A 81: 6993–6997. 6209710 10.1073/pnas.81.22.6993 PMC392062

[18] Barrionuevo F, Scherer G (2010) SOX E genes: SOX9 and SOX8 in mammalian testis development. Int J Biochem Cell Biol 42: 433–436. doi: 10.1016/j.biocel.2009.07.015 19647095 10.1016/j.biocel.2009.07.015

[19] Miyagi S, Kato H, Okuda A (2009) Role of SoxB1 transcription factors in development. Cell Mol Life Sci 66: 3675–3684. doi: 10.1007/s00018-009-0097-0 19633813 10.1007/s00018-009-0097-0 PMC11115863

[20] Uchikawa M, Kamachi Y, Kondoh H (1999) Two distinct subgroups of Group B Sox genes for transcriptional activators and repressors: their expression during embryonic organogenesis of the chicken. Mech Dev 84: 103–120. 10473124 10.1016/s0925-4773(99)00083-0

[21] Fridman JS, Lowe SW (2003) Control of apoptosis by p53. Oncogene 22: 9030–9040. doi: 10.1038/sj.onc.1207116 14663481 10.1038/sj.onc.1207116

[22] Lefebvre V, Dumitriu B, Penzo-Mendez A, Han Y, Pallavi B (2007) Control of cell fate and differentiation by Sry-related high-mobility-group box (Sox) transcription factors. Int J Biochem Cell Biol 39: 2195–2214. doi: 10.1016/j.biocel.2007.05.019 17625949 10.1016/j.biocel.2007.05.019 PMC2080623

[23] Tuck SP, Crawford L (1989) Characterization of the human p53 gene promoter. Mol Cell Biol 9: 2163–2172. 2664471 10.1128/mcb.9.5.2163-2172.1989 PMC363010

[24] Mathelier A, Fornes O, Arenillas DJ, Chen CY, Denay G, Lee J, et al (2016) JASPAR 2016: a major expansion and update of the open-access database of transcription factor binding profiles. Nucleic Acids Res 44: D110–115. doi: 10.1093/nar/gkv1176 26531826 10.1093/nar/gkv1176 PMC4702842

[25] Wang S, El-Deiry WS (2006) p73 or p53 directly regulates human p53 transcription to maintain cell cycle checkpoints. Cancer Res 66: 6982–6989. doi: 10.1158/0008-5472.CAN-06-0511 16849542 10.1158/0008-5472.CAN-06-0511

[26] Deffie A, Wu H, Reinke V, Lozano G (1993) The tumor suppressor p53 regulates its own transcription. Mol Cell Biol 13: 3415–3423. 7684498 10.1128/mcb.13.6.3415-3423.1993 PMC359810

[27] Ashcroft M, Vousden KH (1999) Regulation of p53 stability. Oncogene 18: 7637–7643. doi: 10.1038/sj.onc.1203012 10618703 10.1038/sj.onc.1203012

[28] Shieh SY, Ikeda M, Taya Y, Prives C (1997) DNA damage-induced phosphorylation of p53 alleviates inhibition by MDM2. Cell 91: 325–334. 9363941 10.1016/s0092-8674(00)80416-x

[29] Dulic V, Kaufmann WK, Wilson SJ, Tlsty TD, Lees E, Harper JW, et al (1994) p53-dependent inhibition of cyclin-dependent kinase activities in human fibroblasts during radiation-induced G1 arrest. Cell 76: 1013–1023. 8137420 10.1016/0092-8674(94)90379-4

[30] el-Deiry WS, Tokino T, Velculescu VE, Levy DB, Parsons R, Trent JM, et al (1993) WAF1, a potential mediator of p53 tumor suppression. Cell 75: 817–825. 8242752 10.1016/0092-8674(93)90500-p

[31] Gu W, Roeder RG (1997) Activation of p53 sequence-specific DNA binding by acetylation of the p53 C-terminal domain. Cell 90: 595–606. 9288740 10.1016/s0092-8674(00)80521-8

[32] Harper JW, Adami GR, Wei N, Keyomarsi K, Elledge SJ (1993) The p21 Cdk-interacting protein Cip1 is a potent inhibitor of G1 cyclin-dependent kinases. Cell 75: 805–816. 8242751 10.1016/0092-8674(93)90499-g

[33] Saramaki A, Banwell CM, Campbell MJ, Carlberg C (2006) Regulation of the human p21(waf1/cip1) gene promoter via multiple binding sites for p53 and the vitamin D3 receptor. Nucleic Acids Res 34: 543–554. doi: 10.1093/nar/gkj460 16434701 10.1093/nar/gkj460 PMC1351372

[34] Xiong Y, Hannon GJ, Zhang H, Casso D, Kobayashi R, Beach D (1993) p21 is a universal inhibitor of cyclin kinases. Nature 366: 701–704. doi: 10.1038/366701a0 8259214 10.1038/366701a0

[35] Bargonetti J, Manfredi JJ (2002) Multiple roles of the tumor suppressor p53. Curr Opin Oncol 14: 86–91. 11790986 10.1097/00001622-200201000-00015

[36] Mowat MR (1998) p53 in tumor progression: life, death, and everything. Adv Cancer Res 74: 25–48. 9561266 10.1016/s0065-230x(08)60764-2

[37] Prives C, Hall PA (1999) The p53 pathway. J Pathol 187: 112–126. doi: 10.1002/(SICI)1096-9896(199901)187:1<112::AID-PATH250>3.0.CO;2-3 10341712 10.1002/(SICI)1096-9896(199901)187:1<112::AID-PATH250>3.0.CO;2-3

[38] Luo Q, Beaver JM, Liu Y, Zhang Z (2017) Dynamics of p53: A Master Decider of Cell Fate. Genes (Basel) 8.

[39] Hainaut P, Hollstein M (2000) p53 and human cancer: the first ten thousand mutations. Adv Cancer Res 77: 81–137. 10549356 10.1016/s0065-230x(08)60785-x

[40] Hollstein M, Sidransky D, Vogelstein B, Harris CC (1991) p53 mutations in human cancers. Science 253: 49–53. 1905840 10.1126/science.1905840

[41] Ajay AK, Meena AS, Bhat MK (2012) Human papillomavirus 18 E6 inhibits phosphorylation of p53 expressed in HeLa cells. Cell Biosci 2: 2 doi: 10.1186/2045-3701-2-2 22244155 10.1186/2045-3701-2-2 PMC3285035

[42] Scheffner M, Werness BA, Huibregtse JM, Levine AJ, Howley PM (1990) The E6 oncoprotein encoded by human papillomavirus types 16 and 18 promotes the degradation of p53. Cell 63: 1129–1136. 2175676 10.1016/0092-8674(90)90409-8

[43] Zhang Y, Xiong Y, Yarbrough WG (1998) ARF promotes MDM2 degradation and stabilizes p53: ARF-INK4a locus deletion impairs both the Rb and p53 tumor suppression pathways. Cell 92: 725–734. 9529249 10.1016/s0092-8674(00)81401-4

[44] Miyashita T, Reed JC (1995) Tumor suppressor p53 is a direct transcriptional activator of the human bax gene. Cell 80: 293–299. 7834749 10.1016/0092-8674(95)90412-3

[45] Han F, Liu W, Jiang X, Shi X, Yin L, Ao L, et al (2015) SOX30, a novel epigenetic silenced tumor suppressor, promotes tumor cell apoptosis by transcriptional activating p53 in lung cancer. Oncogene 34: 4391–4402. doi: 10.1038/onc.2014.370 25435374 10.1038/onc.2014.370 PMC4541146

[46] Hur W, Rhim H, Jung CK, Kim JD, Bae SH, Jang JW, et al (2010) SOX4 overexpression regulates the p53-mediated apoptosis in hepatocellular carcinoma: clinical implication and functional analysis in vitro. Carcinogenesis 31: 1298–1307. doi: 10.1093/carcin/bgq072 20400479 10.1093/carcin/bgq072

[47] Wang J, Ding S, Duan Z, Xie Q, Zhang T, Zhang X, et al (2016) Role of p14ARF-HDM2-p53 axis in SOX6-mediated tumor suppression. Oncogene 35: 1692–1702. doi: 10.1038/onc.2015.234 26119940 10.1038/onc.2015.234 PMC4820682

[48] Pan X, Zhao J, Zhang WN, Li HY, Mu R, Zhou T, et al (2009) Induction of SOX4 by DNA damage is critical for p53 stabilization and function. Proc Natl Acad Sci U S A 106: 3788–3793. doi: 10.1073/pnas.0810147106 19234109 10.1073/pnas.0810147106 PMC2656158

