# Supplementary figures and images for: SOX14 activates the p53 signaling pathway and induces apoptosis in a cervical carcinoma cell line
Source: PLoS One. 2017 Sep 19;12(9):e0184686. doi: 10.1371/journal.pone.0184686 (PMC5604970; doi:10.1371/journal.pone.0184686)

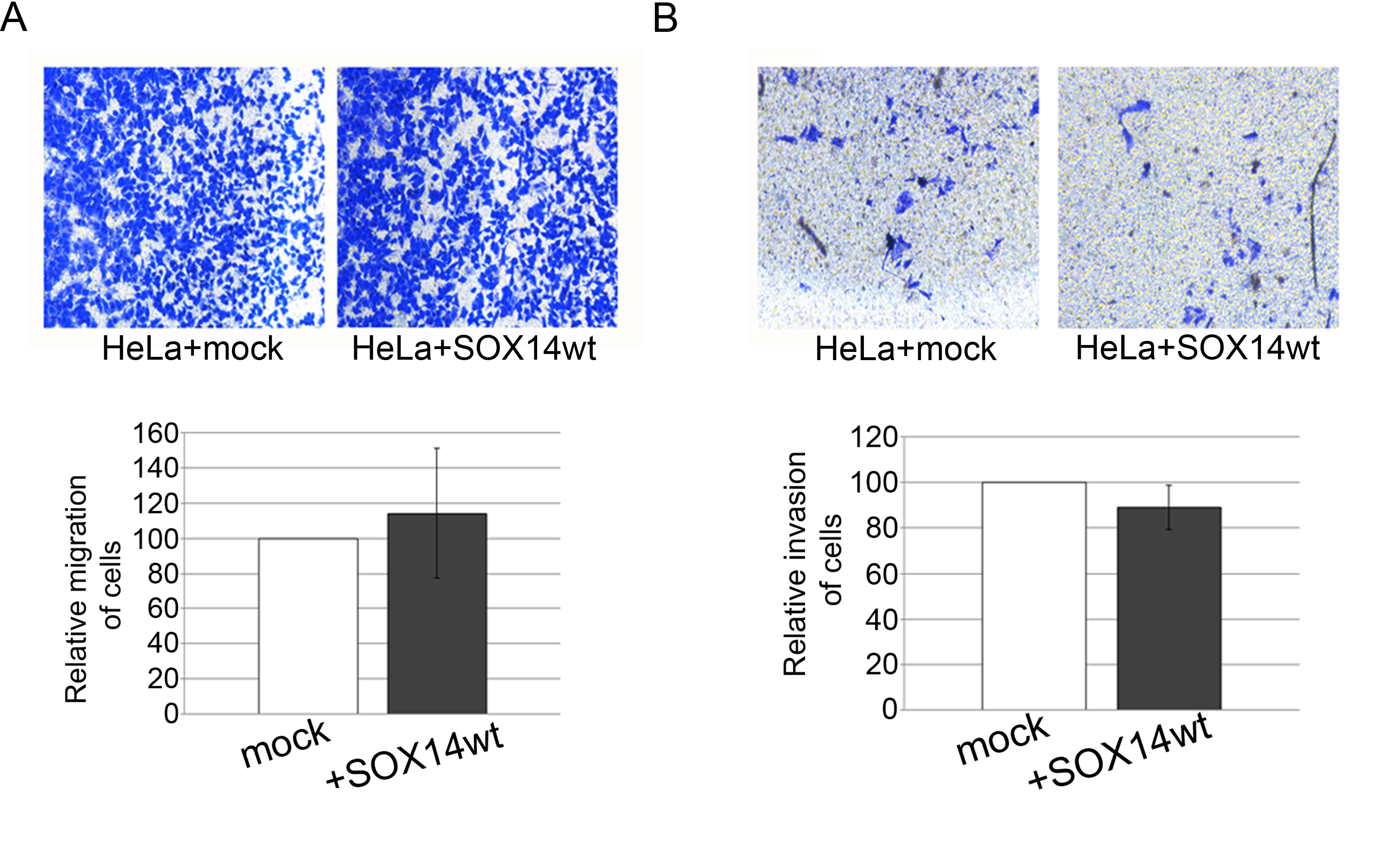

Supplement: S1 Fig — A—Transwell migration assay on HeLa cells transfected with empty vector (mock) or SOX14wt. Representative images of the transwell migration assay are presented. The relative change in cell migration was calculated as a percentage of HeLa cell migration after mock transfection that was set as 100%. Cells were counted from five fields and averages were calculated. Results are presented as the means ± SEM of at least three independent experiments performed in duplicate. B—Transwell invasion assay on HeLa cells transfected with empty vector (mock) or SOX14wt. Representative images of the transwell invasion assay are presented. The relative change in cell invasion was calculated as a percentage of HeLa cell invasion after mock transfection that was set as 100%. Cells were counted from five fields and averages were calculated. Results are presented as the means ± SEM of at least three independent experiments performed in duplicate. (TIF) [file pone.0184686.s001.tif]
